# Stenting of branch pulmonary artery stenosis in children: initial experience and mid-term follow-up of the pul-stent

**DOI:** 10.1007/s00380-023-02246-9

**Published:** 2023-02-23

**Authors:** Xinyi Xu, Ying Guo, Meirong Huang, Lijun Fu, Fen Li, Haibo Zhang, Wei Gao, Tingliang Liu

**Affiliations:** 1 Department of Cardiology, Shanghai Children’s Medical Center, School of Medicine, Shanghai Jiao Tong University, 1678 Dongfang Road, Shanghai, 200127 China; 2 Department of Cardiothoracic Surgery, Shanghai Children’s Medical Center, School of Medicine, Shanghai Jiao Tong University, 1678 Dongfang Road, Shanghai, 200127 China

**Keywords:** Pulmonary artery stenosis, Stent implantation, Redilation, Intervention

## Abstract

Not all stents are suitable for children. For instance, premounted stents can be used in infants and small children but cannot dilate with age to accommodate adult-sized pulmonary arteries. Conversely, the Pul-Stent adapts to somatic growth. Thus, our hospital implemented the Pul-Stent in pediatric patients with branch pulmonary artery stenosis. This study summarizes our initial experience with Pul-Stents in this patient population, including the efficacy and safety. We implanted 37 Pul-Stents in 35 patients between August 2014 and June 2015. The patients’ mean age and weight at stent implantation were 6.7 ± 3.0 years and 20.9 ± 8.7 kg, respectively. Bench testing revealed that axial shortening of the Pul-Stent was minimal with further dilation, and the radial strength did not change. The stents were successfully deployed in all cases, except two with minor malpositioning. Primarily, 8–12 mm mounting balloons were used for the initial implantation, and a long sheath (8–10 F) was used for delivery. After stent implantation, the minimal lumen diameter in the stenosed segment increased by 50% in 97% (34/35) of patients. Furthermore, the pressure gradient across the stenosed segment decreased by 50% in 77% (23/30) of biventricular patients. One stent fracture and one stent restenosis were noted during the follow-up visits (mean follow-up time: 4.6 ± 1.7 years). Eighteen patients (51%) underwent repeat catheterization; ten had successful redilation. No aneurysms or stent fractures were observed. Our initial results indicate that the Pul-Stent is safe and effective in pediatric patients and can be further dilated over time to accommodate somatic growth. Moreover, the Pul-Stent has good compliance and adequate radial strength to treat pulmonary artery stenosis effectively.

## Introduction

In modern times, intravascular stent implantation has become the preferred treatment for most branch pulmonary artery stenosis (PAS) cases. Stents provide a radial force that prevents elastic recoil and is more effective for long-term obstruction relief than balloon angioplasty alone [1]. Additionally, numerous reports have demonstrated the safety and efficacy of stent implantations, reporting excellent outcomes for PAS [2].

Stents for treating PAS are mainly used off-label despite several available options. Furthermore, most of these stents are not marketed in China. As of 2022, only NuMED Cheatham-Platinum (CP) and premounted stents, such as the Cordis Plamaz Blue and Cordis Plamaz Genesis, are available for PAS in China. CP stents are specifically designed to treat vascular obstructions associated with congenital heart disease (CHD) and have diameters that expand from 12 to 24 mm, allowing for somatic growth. However, this requires larger delivery sheaths (10–12 F), limiting their use in young children [3]. Premounted stents are designed for adult coronary and peripheral vessels but have small- to medium-sized diameters, which is advantageous for relieving obstruction and immediately improving hemodynamics in infants and small children. However, they cannot dilate as the child ages to accommodate adult-sized pulmonary arteries up to 20 mm and have high rates of transcatheter and surgical reinterventions [4].

**Fig. 1:**
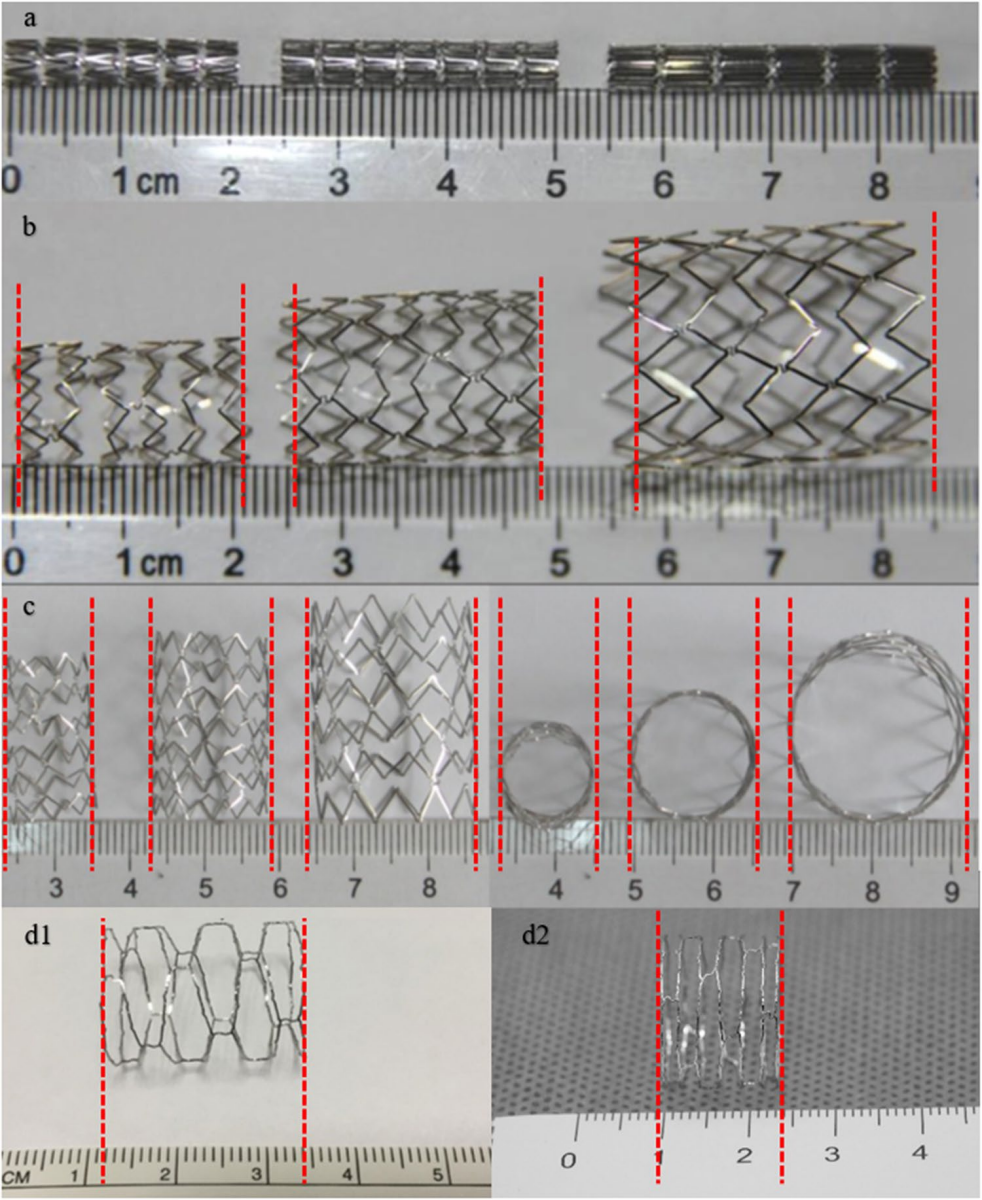
Pul-Stent bench testing and dilations. **a** From left to right: S, M, and L Pul-Stents with unexpanded lengths of 20, 25, and 30 mm, respectively. **b**–**c** From left to right: S, M, and L Pul-Stents expanded to their maximal shaft diameters of 12, 16, and 22 mm, respectively. The length of model S did not considerably decrease, and the axial shortening rates of models M and L were < 15%. d1: Mild axial shortening occurred in model S (unexpanded length: 25 mm) after overexpanding with 18 mm × 25 mm high-pressure balloons. d2: Considerable radial shortening (40%) occurred in model S (unexpanded length: 25 mm) after overexpanding with 18 mm × 40 mm high-pressure balloons

The Med-Zenith Pulmonary Artery Stent (Pul-Stent; Med-Zenith Medical Scientific Co., Ltd, Beijing, China) is a laser engraved stent made from cobalt-based (L605) alloy tubing and licensed by the China National Medical Products Administration in 2020 specifically for the treatment of congenital or acquired PAS. This endovascular stent is an unmounted balloon-expandable stent with a low crimping profile and high radial force (mean stent recoil, 2.5%). Furthermore, it has good X-ray visibility and magnetic resonance compatibility owing to the cobalt alloy properties and semi-open cell design. Additionally, a small delivery sheath is used for implantation, and the axial shortening after re-dilation is less than 20%. The Pul-Stent is available in three models: S (6–12 mm), M (12–16 mm), and L (18–22 mm; Fig. 1). The nominal unexpanded length also varies from 15 to 40 mm in 5 mm increments. However, only model S stent has the shortest nominal unexpanded length of 15 mm, whereas the unexpanded lengths of model M and L stents begin at 20 mm. After expanding to the maximum diameter according to the manufacturer (12, 16, and 22 mm for models S, M, and L, respectively), the foreshortening of model S ranges from 2.7 to 16.7%, model M from 6.8 to 9.2%, and model L from 13.9%to 15.3%. Therefore, the Pul-Stent may benefit children with PAS by allowing re-dilation for somatic growth without significant foreshortening and avoiding jailing a significant side branch.

Our hospital tested the use of Pul-Stents in pediatric patients with congenital or postoperative branch PAS. Therefore, this report summarizes our initial experience and assesses immediate and mid-term outcomes.

## Materials and methods

### Patients

We enrolled consecutive pediatric patients who underwent Pul-Stent implantation at our institution from August 2014 to June 2015. We implanted 37 Pul-Stents in 35 patients with congenital or postoperative PAS. Then, we retrospectively reviewed their medical records for demographic data and clinical characteristics, including previous surgical and catheter interventions and follow-up information. The Ethics Committee of the Shanghai Children’s Medical Center authorized all protocols and procedures in this study (SCMCIRB-W2021050), and each patient’s guardian or the patient (when applicable) gave their informed consent for inclusion before they participated in the study. All procedures performed in this study were in accordance with the relevant guidelines and regulations and with the 1964 Helsinki declaration and its later amendments or comparable ethical standards.

### Bench testing

Bench testing was performed using a sample stent design. First, all three stent sizes were inflated and expanded to their nominal maximal diameter, as recommended by the manufacturer. The other two model S stents were then over-dilated using 18-mm high-pressure balloons with different lengths. Next, the shortening characteristics, stent integrity (e.g., strut design and fractures), and stent length were measured at each sequential dilation stage. The BIB balloon (NuMED, Hopkinton, NY, USA) and the Atlas Gold high-pressure balloon (Bard Peripheral Vascular, Tempe, AZ) were used for dilation and overdilation, respectively.

### Indications

Pulmonary stent implantation was indicated if at least one of the following was present [5]: (1) a measurable pressure gradient greater than 20 mmHg across the stenosis area or a right-ventricular pressure greater than one-half to two-thirds of the systemic pressure in a biventricular physiology; (2) a blood flow discrepancy between the lungs of 35%/65% or worse identified by cardiac magnetic resonance imaging; and (3) significant narrowing (> 50%) of the adjacent vessel for single-or biventricular patients identified by cardiac angiography.

### Procedures

All catheter procedures were performed under general anesthesia with endotracheal intubation. Diagnostic catheterization was conducted to evaluate hemodynamic and morphological data using the femoral or right internal jugular vein as access vessels. An appropriate stent was chosen based on the results of the angiography measurements. Before insertion, the Pul-Stent was manually crimped onto a balloon, the size of which did not exceed the diameter of the segment adjacent to the narrowing segment. Importantly, short and non-compliant balloons were used to prevent “dog boning.” After the stents were delivered to the stenotic lesion, angiography was performed to confirm the correct position. Then, the balloon was inflated to expand the stent to the desired diameter. Acute outcomes were evaluated using repeat angiography and hemodynamic measurements. All patients began a 3–5 mg/kg/day (maximum 100 mg/day) dose of aspirin, administered for 6 months after the procedure. Additionally, advanced anticoagulation (warfarin) was prescribed to patients with univentricular circulation.

### Follow-up protocol

All patients underwent clinical assessment and transthoracic echocardiography (TTE) the day after the procedure. Chest radiographs (anteroposterior and lateral views) were also obtained before discharge three to five days after the procedure. Following hospital discharge, repeat radiography and TTE evaluations were scheduled after 1, 6, and 12 months, and then yearly after that. In addition, repeat catheterization was performed if the TTE indicated that the pressure gradient across the stent was 50% higher than that immediately after surgery.

### Statistical methods

Continuous variables were described as means ± standard deviations for normally distributed data or medians (ranges) for non-normally distributed data. Categorical variables were described as counts and percentages. Outcome measures (e.g., a decreased systolic pressure gradient across the pulmonary artery stenosis, increased stented vessel diameter in pulmonary artery stenting, and decreased superior vena cava pressure in cavopulmonary anastomosis) were assessed and compared pre-and postintervention. Comparisons between the groups were performed using a two-tailed t test or Wilcoxon rank-sum test. All analyses were performed using SPSS software for Windows (version 26.0, IBM Corp., Armonk, NY, USA). A *P* value of < 0.05 was considered statistically significant.

## Results

### Bench testing

Pul-Stent models S, M, and L with unexpanded lengths of 20, 25, and 30 mm, respectively, were used for bench testing. After expanding to the maximum shaft diameter recommended by the manufacturer (12, 16, and 22 mm for models S, M, and L, respectively), model S maintained its pre-expanded length, and the axial shortening rates of models M and L were less than 15% (Fig. 1a–c). Next, 18 mm × 25 mm and 18 mm × 40 mm high-pressure balloons were used to over-dilate the other two model S stents with unexpanded lengths of 25 mm. A mild radial shortening of the stent (15%) occurred after dilating with 25 mm-length balloon (Fig. 1d1), but considerable radial shortening (40%) occurred with 40 mm-length balloon (Fig. 1d2).

### Demographic data

The study included 14 boys and 21 girls who underwent Pul-Stent implantation. The mean age and weight at stent implantation were 6.7 ± 3.0 years and 20.9 ± 8.7 kg, respectively. Thirty-three patients (33/35, 94.3%) underwent postoperative CHD repair; five of whom underwent single-ventricle repair (bidirectional Glenn = 3, Fontan = 2) for complex CHD. Post-pulmonary atresia with ventricular septal defect (37.1%) and tetralogy of Fallot (31.4%) were the most common underlying heart diseases. One patient had ventricular septal defects that spontaneously closed before stent implantation. Another patient had isolated branch pulmonary stenosis without other congenital heart diseases. Percutaneous stent implantation was displacement performed in all patients except one who received hybrid stent implantation in the operating room under direct visualization.

In total, 37 Pul-Stents were implanted (model *S* = 20, model *M* = 16, and model *L* = 1). The stents were implanted in the proximal left pulmonary artery (*n* = 24) and the proximal right pulmonary artery (*n* = 13). Table 1 presents the patients’ demographic and clinical characteristics.

**Table 1:** The demographic and procedural data of the patients with Pul-Stent stenting

No	Weight (kg)	Age (yrs)	Biventricle/single ventricle	Stent (model. length)	Target lesion	Used balloon	Sheath size	Size of balloon (mm)	Stenosis (pre)	Stenosis (post)	Follow-up (yrs)	Complication
1	20.7	7.8	Single ventricle	S25	LPA	Z-MED II	10F	8 × 30	0.0	7.5	2.6	Restenosis
2	21.8	7.6	Biventricle	M20	LPA	BIB	12F	16 × 45	7.4	16.2	5.7	
3	25.0	7.9	Biventricle	S25	LPA	Z-MEDII	8F	10 × 30	4.0	8.5	6.1	
4	31.7	13.6	Biventricle	M25	LPA	Z-MEDII	8F	12 × 30	3.7	10.8	1.1	
5	13.0	3.4	Biventricle	M20	LPA	Z-MEDII	9F	10 × 30	3.3	7.8	4.0	Hemorrhage
6	19.0	7.0	Single ventricle	S20	LPA	EV3	8F	6 × 30	0.0	6.0	3.9	
7	22.0	7.6	Single ventricle	M20	LPA	Z-MEDII	9F	10 × 30	6.4	8.5	5.7	
8	15.5	6.5	Biventricle	M25	LPA	EV3	/*	10 × 30	4.3	8.4	5.0	
9	32.3	10.4	Biventricle	M25	LPA	EV3	10F	10 × 30	4.5	10.2	5.4	
10	25.4	9.2	Biventricle	M25	RPA	Z-MEDII	9F	10 × 30	5.6	10.9	4.5	
11	29.0	9.8	Biventricle	M20	RPA	EV3	10F	12 × 20	6.4	11.7	1.9	
12	11.3	4.3	Biventricle	S15	LPA	EV3	8F	8 × 30	4.9	7.3	4.9	
13	17.4	5.2	Biventricle	S15	LPA	EV3	8F	8 × 30	4.8	8.0	3.7	
14	27.6	10.7	Biventricle	M20	LPA	EV3	/*	12 × 30	3.0	9.0	5.8	
15	17.6	6.2	Biventricle	S20	LPA	EV3	8F	8 × 30	2.4	7.3	6.7	
16	14.9	4.1	Biventricle	S20/M20	LPA	EV3/EV3	9F	10 × 30/10 × 30	3.8	9.2	5.0	Malposition
17	18.0	6.5	Biventricle	S15	RPA	EV3	9F	12 × 40	4.3	8.3	4.8	
18	29.5	11.4	Biventricle	M25	RPA	EV3	10F	12 × 40	6.0	11.2	5.5	
19	13.0	3.3	Biventricle	S20	LPA	EV3	8F	8 × 30	2.3	5.2	6.4	Hemorrhage
20	13.8	4.6	Biventricle	S20	LPA	EV3	8F	10 × 30	4.0	9.0	2.3	
21	15.0	2.3	Biventricle	S20	RPA	EV3	7F	8 × 30	2.0	8.0	5.5	
22	19.9	9.1	Biventricle	L20	LPA	Z-MEDII	10F	12 × 20	4.2	9.2	2.5	
23	18.8	6.0	Biventricle	M25	RPA	Z-MEDII	9F	12 × 30	6.3	11.3	5.3	
24	23.0	9.0	Biventricle	S20	LPA	EV3	8F	8 × 30	2.3	7.6	5.5	
25	17.8	4.3	Single ventricle	S15	RPA	EV3	8F	8 × 30	2.2	7.0	5.5	
26	15.1	3.3	Biventricle	S20	RPA	EV3	8F	10 × 30	4.0	8.0	6.0	
27	12.3	2.9	Biventricle	S15	LPA	EV3	8F	10 × 30	4.2	8.8	5.0	
28	52.0	9.3	Biventricle	M25/M25	RPA	EV3/Z-MEDII	10F	12 × 40/16 × 30	6.5	13.4	6.8	Malposition
29	13.2	3.2	Biventricle	S15	RPA	EV3	8F	8 × 30	3.6	7.5	1.2	
30	18.0	4.9	Biventricle	S20	LPA	EV3	8F	8 × 30	3.5	8.9	4.3	
31	19.1	6.4	Biventricle	S15	LPA	EV3	10F	8 × 30	3.5	7.5	4.4	
32	22.3	4.6	Biventricle	S20	LPA	EV3	8F	10 × 30	3.6	8.3	5.3	
33	41.0	13.5	Biventricle	M20	RPA	EV3	8F	10 × 30	4.8	11.3	3.3	
34	13.4	2.8	Biventricle	S15	LPA	EV3	8F	8 × 30	3.7	6.9	5.0	
35	12.7	5.2	Single ventricle	S15	LPA	EV3	8F	8 × 30	2.6	8.0	1.1	

*LPA* left pulmonary artery; *RPA* right pulmonary artery

*Two patients underwent Pul-Stent implantation during surgery repair without an introducer sheath

### Immediate results

The stents were successfully deployed in all cases, except two with minor malpositioning after an expansion that did not completely cover the PAS. However, these cases were resolved by placing a second stent during the same procedure. Balloon ruptures, stent on the balloon, and considerable stent embolization did not occur during the implantation procedures. Self-limited pulmonary hemorrhage occurred in two patients, possibly secondary to tiny vascular ruptures, an acute increase in perfusion to the affected segment, or guidewire trauma. The minimum shaft dimensions of the balloons used to crimp the stents were 6 mm for model S, 10 mm for model M, and 12 mm for model L, and the median balloon diameter at the initial dilation was 10.0 mm (range, 6–16.0 mm).

**Fig. 2:**
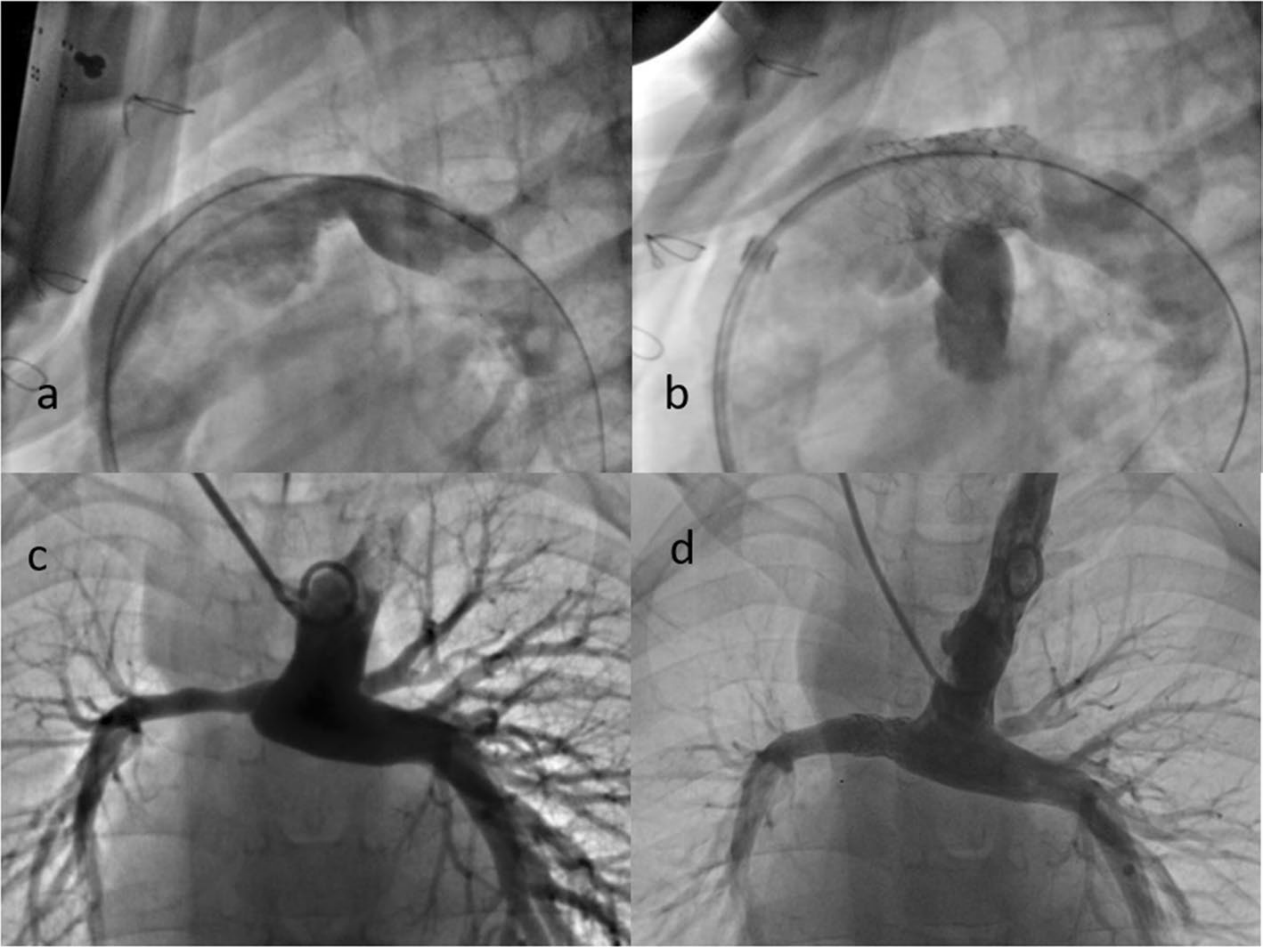
Representative images of stenting with Pul-Stents. Left pulmonary artery stenosis (**a**) before and **b** after stenting in a 7-year-old boy (weight: 21 kg) with postoperative status of tetralogy of Fallot (model M with 16 mm BIB balloon). Right pulmonary artery stenosis (**c**) before and (**d**) after stenting in a four-year-old girl (weight: 17 kg) who underwent a Glenn procedure (model S with 10 mm EV3 balloon)

After stent implantation, 97% (34/35) of patients had a 50% increase in the minimal lumen diameter of the stenosed segment (Fig. 2), and 77% of biventricular patients (23/30) had a 50% decrease in the pressure gradient across the stenosed segment. Overall, the mean minimum diameter of the pulmonary artery stenosis increased from 3.9 ± 1.7 mm to 8.9 ± 2.1 mm (*P* < 0.001). Among the biventricular patients (*n* = 30), the mean systolic pressure gradient across the pulmonary artery stenosis decreased from 30.1 ± 12.2 to 9.7 ± 9.5 mmHg (*P* < 0.001), and the right ventricular-to-aortic pressure ratio decreased from 0.57 ± 0.14 to 0.43 ± 0.12 (*P* < 0.001). For patients with a postoperative cavopulmonary anastomosis (*n* = 5), the mean superior vena cava pressure after stenting decreased from 17.0 ± 1.9 to 14.0 ± 0.7 mmHg (*P* = 0.046).

### Follow-up results

All patients underwent serial clinical assessments and imaging evaluations at least 1 year after stent implantation. The longest follow-up was 7.5 years, with a mean follow-up of 4.6 ± 1.7 years. One stent fracture (axial fracture occurring through stent nodes) was noted on a chest X-ray 5 years after implanting the stent. Two patients died 13 and 14 months after the stenting procedure from surgical complications during the repair of additional defects; their deaths were unrelated to the stent. All other patients were clinically stable throughout the follow-up, and no deaths were attributed to stent implantation.

Eighteen patients (51%) had repeat catheterization after a mean period of 3.6 ± 2.1 years after the initial implantation. Obvious side-branch vessel jailing or evidence of aneurysm formation was not identified, except for one patient with significant in-stent restenosis. During the follow-up, the stent’s minimal diameter did not differ from the diameter at implantation (follow-up: 8.5 ± 2.8 mm vs. initial: 8.9 ± 1.9 mm,* P* = 0.348, *n* = 18). However, the gradient in biventricular patients was slightly higher at the follow-up visit than initially (follow-up: 21.1 ± 18.1 mmHg vs. initial: 10.1 ± 10.4, *P* = 0.015, *n* = 14).

**Fig. 3:**
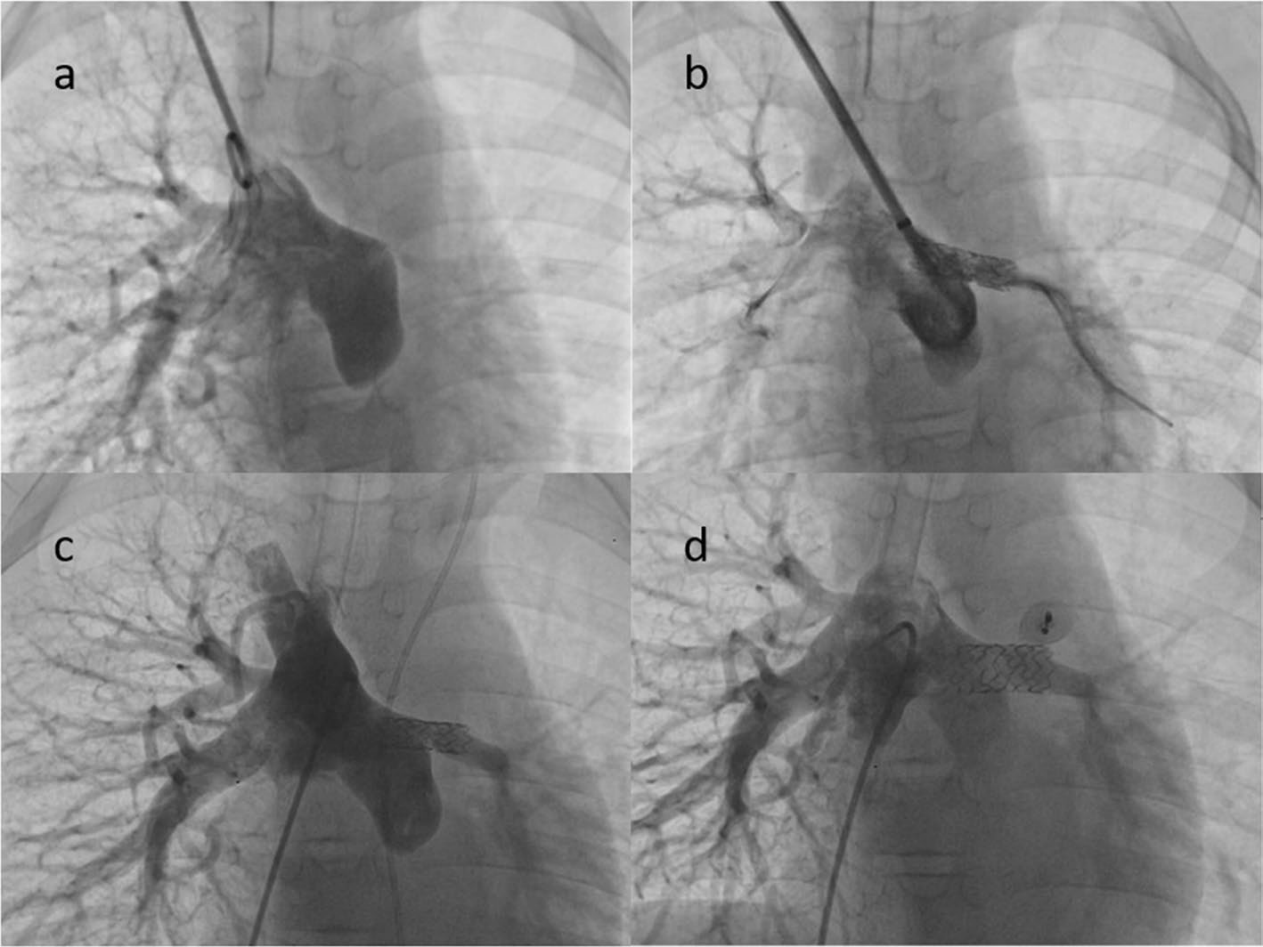
Stenting a left pulmonary artery (LPA) stenosis with Pul-Stents in a 6-year-old boy (weight: 19 kg) after a Fontan procedure. **a** A severe LPA stenosis with atresia tendencies. **b** Initial stenting with the Pul-Stent (model S) with a 6-mm EV3 balloon after sequential dilation with a series of coronary balloons. **c** Angiography at the 4-year follow-up did not identify stent stenosis, distortion, or fracture. **d** Further dilation of the stent with a 10-mm EV3 balloon resulted in mild stent shortening without stent fractures or migration

Ten patients underwent stent re-dilation (model *S* = 9, model *M* = 1) after a mean follow-up of 3.8 ± 2.0 years after implantation, improving the stent diameter from, on average, 7.0 ± 2.7 mm to 10.8 ± 1.6 mm (*P* < 0.001). Furthermore, in the biventricular patients (n = 7), the gradient pressure changed, on average, from 25.0 ± 18.3 mmHg to 7.3 ± 4.9 mmHg (*P* = 0.036). Repeat dilations were primarily indicated for arterial segments with a larger diameter or residual waist, except for one patient with an occlusive left pulmonary artery after the Glenn procedure; this patient had significant intimal proliferation and severe in-stent stenosis at the 1-year follow-up. Furthermore, despite receiving aspirin and warfarin after the initial procedure and a successful re-dilation, the stent became obstructed again, but a third intervention was abandoned. Stent fractures or migrations were not observed on further dilation (Fig. 3).

**Fig. 4:**
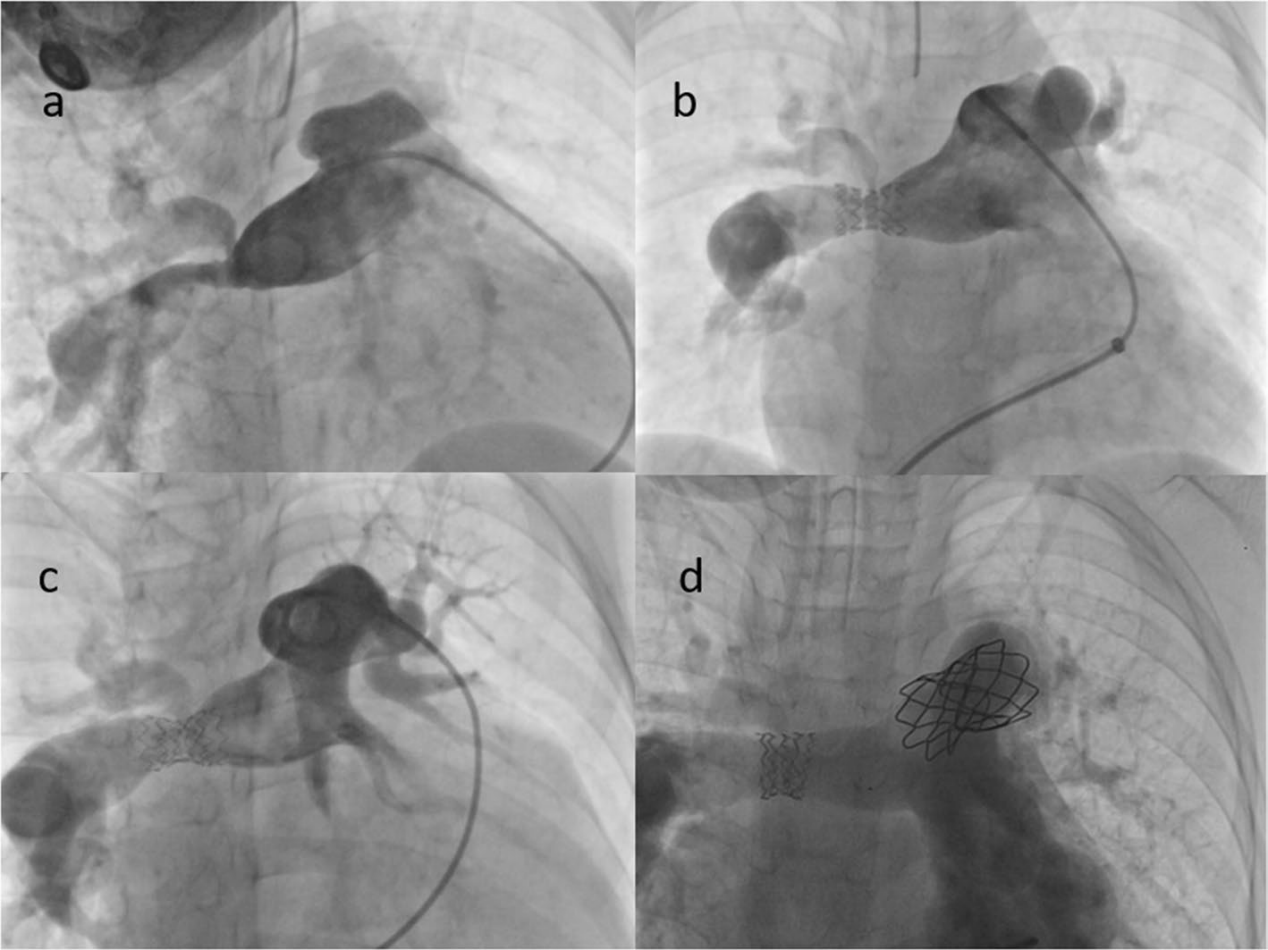
Stenting of a right pulmonary RPA stenosis in a 6-year-old boy (weight: 18 kg) after the surgery of tetralogy of Fallot. **a** A severe stenosis of the RPA bifurcation. **b** Initial stenting with a Pul-Stent (model S, length: 15 mm) with a 12-mm EV3 balloon without jailing the right upper pulmonary artery branch. **c** Angiography at 4-year follow-up did not identify stent stenosis or fracture, and the right upper pulmonary artery branch still filled freely through the side of the stent. **d** A “dog boning” stent with significant shortening after further dilation with a 15 mm × 40 mm non-compliant balloon and a 14 mm × 40 mm high-pressure balloon for the resistant stenosis. Also, a Cheatham-Platinum stent is present in the left pulmonary artery, implanted during a previous surgical repair

One model S stent with an unexpanded length of 15 mm had significant shortening of the overall length after re-dilation with an oversized non-compliant balloon (15 mm × 40 mm) and a high-pressure balloon (14 mm × 40 mm) for a resistant residual waist. Nonetheless, it covered the entire lesion with a good radial force and no recoil (Fig. 4).

### Discussion

PAS is common when treating children with various congenital heart diseases and genetic syndromes, such as Williams syndrome, Alagille syndrome, and Noonan syndrome. In the current era, transcatheter interventional strategies are the standard treatments for native and postoperative PAS, since surgical repair presents several challenges, such as reaching distal stenoses, high restenosis rates, and discouraging effects with multiple lesions. Mullins et al. first described endovascular stenting for CHD in 1988 [6]. Since then, the materials and manufacturing technologies have rapidly developed, making stent implantation an effective solution for treating PAS of various morphological types.

Balloon angioplasty is relatively easy to perform and requires minimal vascular access. However, it is not ideal for resistant stenosis, distally located PAS, lesions secondary to external compression, and long-segment hypoplasia [7]. For these lesions, stent implantation may help achieve long-lasting enlargement to the desired diameter by exerting a radial force that prevents elastic recoil. Additionally, stenting at a younger age improves proximal and distal pulmonary perfusion and promotes lobar branch growth in single-and biventricular patients [8]. However, pulmonary artery stent implantation in small and hostile structures still has challenges, such as stent inflexibility, a requirement for large sheaths, and stent expansion to match a child’s somatic growth.

This study investigated the efficacy and safety of Pul-Stents for treating pediatric patients with branch PAS. Immediately after stent implantation, 97% of the patients (34/35) had a 50% increase in the minimal lumen diameter of the stenosed segment, and 77% (23/30) of biventricular patients had a 50% decrease in the pressure gradient across the stenosed segment. In our initial experience, the Pul-Stent which had hybrid-cell design and “S” shaped poly-links provided good tracking and delivery performance with adequate radial support while allowing serial re-dilation to accommodate somatic growth with minimal shortening. The edges of the Pul-Stent are round and smooth, which minimizes the potential for luminal trauma during balloon expansion. Furthermore, the Pul-Stent has three models with a wide selection of diameters and lengths, making them appropriate for every possible localization. Finally, the hybrid-cell design of the Pul-Stent allows for branched vessel crossing and dilation of jailed side branches, commonly encountered in pulmonary artery stent implantation.

Since the strut thickness of the Pul-Stent is 0.25 mm for model S and 0.28 mm for models M and L, respectively, the manufacturer recommends using a long sheath two sizes larger than the desired balloon catheter for all three model stents. However, this can be adaptable in practical application because of the numerous types of balloons. In a few cases in this study as well as in other patients, we successfully implanted Pul-Stent using a long sheath that was only one size larger than the mounting balloon (e.g., a 7F sheath for S stent loaded on an 8 mm balloon and an 8F sheath for M stent loaded on a 12 mm balloon) after testing their performance outside the body. Additionally, the manufacturer recommends implanting the Pul-Stent by mounting it on a “Z-MED II” balloon when the outer diameter of the pulmonary artery stent (especially the model M stent) after dilation is less than 10 mm. However, EV3 balloons (8–10 mm) are commonly used during Pul-Stent implantations at our institution, sometimes resulting in an insufficiently stable stent and balloon movement. In our experience, minimally dilating the balloon resulted in a more stable stent. Thus, all Pul-Stent implantations using 8-or 10-mm EV3 balloons in this study were successful, including one patient treated with a 6-mm EV3 balloon to mount a model S stent.

Overall, the mid-term follow-up results have been favorable, and further dilations were safe and effective. One patient had a stent fracture without collapse (identified during a follow-up visit), and one had significant in-stent stenosis after a right-side Glenn procedure, which may have been secondary to the peculiarity of the vascular anatomy. Nonetheless, the Pul-Stent maintained radial force and uniformly expanded with only mild foreshortening and fracture resistance during re-dilation, even when over-dilated. We also used Pul-Stent to treat main pulmonary artery stenosis (not in this study), where the model M stent can be reliably expanded beyond the manufacturer's recommended size to 22 mm without compromising structural integrity (results not shown). However, the stent’s semi-open cell design can result in a mildly irregular shape with small angulation between different struts when it is dilated beyond the maximum diameter recommended by the manufacturer. Nevertheless, both in vitro and in vivo, we found that expanding with balloons appropriately sized to the stent (e.g., not too long) was essential; an inappropriate length can cause substantial stent shortening due to the semi-open cell design, even with high-pressure balloons (e.g., the Atlas Gold balloon). If limited balloon options are available, the lesion’s length and stent shortening should be fully considered before stent expansion with a long-mounting balloon.

Several balloon-expandable stents are commonly used in PAS treatment, such as the Genesis XD stent (Cordis, Fremont, CA, USA) and the IntraStent LD family of stents (Medtronic, Minneapolis, MN, USA), which have properties similar to the Pul-Stent [9]. Besides, Pul-Stent may have minimal clinical significance in infants, where other premounted stents, such as the Valeo stent (Bard Inc., Tempe AZ, USA; low radial strength) and the Formula stent (Cook Medical, Bloomington, IN, USA) could be placed through ≤ 7 F platforms and over-dilated up to adult vessel size to accommodate somatic growth[10, 11]. However, all the stents that are currently on the market have their limitations, including stainless-steel materials, sharp edges, significant foreshortening, and limited radial strength at large diameters, as well as other concerns [12–14]. There is no stent that meets all the requirements for stent implantation in children with congenital heart disease. Furthermore, these aforementioned alternatives are not available in China. Pul-Stent presents a new type of stent available to interventional cardiologists in China, which has a wide range of expansion diameters and lengths, good biocompatibility, and the ability to re-dilation with children’s growth. It is semi-open cell design and “S” shaped hinges provide high trackability and flexibility to the curved vessel geometry with mild foreshortening and sufficient radial strength.

This study was a retrospective review of our experience at a single center. Thus, a small number of patients with different cardiac malformations were included. Also, more than half of our patients underwent stenting with model S; only one required model L. Furthermore, at the time of this study, only half of the patients had undergone repeat catheterization, and less than a quarter of patients had stent re-dilation. Therefore, we cannot present more evidence related to stent foreshortening at the maximal diameter or the properties of large stents when fully expanded in vivo. Furthermore, the follow-up time is insufficient for us to overexpand these stents, particularly model S stent, to near adult pulmonary artery size, as we did in vitro. A larger series of patients with a more extended follow-up period is needed to understand the results of Pul-Stent implantation for PAS.

## Conclusion

The immediate and mid-term follow-up results indicate that Pul-Stents perform well, expanding the spectrum of stents available for treating PAS. This new stent can be further dilated over time to accommodate somatic growth with adequate radial strength while maintaining its integrity. In addition, it is suitable for a wide selection of lengths for every possible localization. However, the lesion length and stent shortening should be fully considered before expanding the stent with a long-mounting balloon because of the stent’s semi-open cell design.

## Data Availability

The original data presented in this study are included in the article, further inquiries can be directed to the corresponding author.
